# “Broken”—How Identities as Women, Mothers and Partners Are Intertwined with the Experience of Living with and Seeking Treatment for Pelvic Organ Prolapse

**DOI:** 10.3390/ijerph19095179

**Published:** 2022-04-24

**Authors:** Kaylee Ramage, Ariel Ducey, Natalie V. Scime, Erin Knox, Erin A. Brennand

**Affiliations:** 1 Department of Obstetrics & Gynecology, Cumming School of Medicine, University of Calgary, 2500 University Drive NW, Calgary, AB T2N 1N4, Canada; natalie.scime@ucalgary.ca (N.V.S.); erin.brennand@albertahealthservices.ca (E.A.B.); 2 Department of Sociology, Faculty of Arts, University of Calgary, 2500 University Drive NW, Calgary, AB T2N 1N4, Canada; aducey@ucalgary.ca (A.D.); erin.knox@ucalgary.ca (E.K.); 3 Department of Community Health Sciences, Cumming School of Medicine, University of Calgary, 2500 University Drive NW, Calgary, AB T2N 1N4, Canada

**Keywords:** pelvic organ prolapse, gendered health behavior, gender equity, gender roles, women’s health

## Abstract

Pelvic Organ Prolapse (POP) occurs when one or more pelvic organs descend into or through the vaginal opening, significantly impacting physical and mental health. POP affects the female reproductive tract and, overwhelmingly, people who identify as women. However, little research has examined the impact of gendered expectations on women’s treatment-seeking for POP and their decision-making around surgery for POP. To address this gap, we conducted semi-structured interviews with 26 women seeking surgery for POP in Alberta, Canada. Data were analyzed from a gender-based lens, using the Framework Method. Participants reported the need to balance their identities as women, partners, and mothers in their pursuit of treatment and faced many barriers to treatment related to their gendered responsibilities. Findings highlight the gendered experiences of prolapse in the context of healthcare needs and can inform policies and practices which promote more equitable access to prolapse treatment.

## 1. Introduction

Over the past several decades, women’s rights in health and healthcare have been evolving, with the promotion of women’s reproductive rights, increased patient involvement in healthcare decision-making, and increased understanding of the intersection between gender and other social determinants of health [1,2]. However, it is clear that gender disparities in health persist for women, and women’s health is influenced by the gendered expectations and limitations within society [3].

Gender is a widely recognized social determinant of health and may be defined as an “individual’s socially ascribed attributes, roles, responsibilities, and expectations in a given society, based on their gender expression and how others perceive it” [4]. Distinct but related to biological sex, which is a product of one’s chromosomes, hormones, reproductive organs, and genitalia, gender is a social construct which recognizes the impact of social systems and processes on the differential distribution of resources and power to people [5], and exists on a continuum. Gender intersects with other dimensions of society, including sexism, the patriarchy, racism, and classism, which ultimately influences the structural pathways leading to health outcomes [6].

Gender can affect health through multiple pathways, including differential exposure to risks, gendered behaviors, healthcare accessibility, and gender bias [6]. The impact of gender on health can range from clearly observable to invisible or unspoken, which can be problematic as people seek care for their health issues.

In general, gender norms constrain women’s power and their ability to make decisions about their own health [7,8,9], which can result in a reality where women are not equal in their ability to choose surgical treatment [10]. Women may postpone treatment due to familial responsibilities, instead prioritizing their children and partners to receive necessary care [11]. Thus, the societal expectations of a “good” mother putting her family first, even if it means deprioritizing her own needs, may impact women’s decision-making around their own health and quality of life (QOL) [12,13]. Because women receive both social and personal affirmation for adhering to these societal ideals [14,15,16], disruption of these norms when a woman develops a health condition and pursues treatment for it can affect their sense of identity as a mother, partner, or “good” woman. For example, an analysis of mothering while living with incurable cancer found that the expectation of motherhood as altruistic pressured women to make decisions in the best interest of their children and partners instead of themselves [17].

Despite the importance of considering gender in women’s health decision-making, there has been limited research on the impact of women’s gendered expectations and responsibilities on their experience of prolapse and their treatment decision-making regarding prolapse. To respond to this need, the current study explored how women navigate their multiple and intersecting gender roles in the context of prolapse and prolapse surgery.

### 1.1. Pelvic Organ Prolapse

Pelvic Organ Prolapse (POP) results from structural changes in the female pelvis that occur during childbirth and/or increasing age. It can occur almost immediately after childbirth, or decades after, and will affect 50% of parous females in their lifetimes [18,19]. When POP is present, one or more pelvic organs (i.e., the uterus, bladder, small bowel, rectum) descend from their normal position to bulge into or through the vaginal opening [20]. POP’s main physical symptom is the sensation of pressure in the pelvis and/or vagina but can also impact women’s lives through associated incontinence, pelvic pain, impairment of sexuality, and negative impacts on mental health and QOL [21,22,23].

### 1.2. Treatment for Pelvic Organ Prolapse

Those experiencing POP have several options for treatment. Non-invasive treatment options include vaginal pessaries or pelvic floor physiotherapy. Pessaries are passive mechanical devices that fit into the vagina, designed to hold the prolapsed organs in their anatomically correct position [24], and are a common first-line option for non-surgical management of POP [25]. Although there are few contraindications to pessary use, a recent Cochrane review showed mixed results about the effectiveness of pessary use on the improvement of prolapse symptoms [24]. Pelvic floor physiotherapy has been shown to have positive impacts on prolapse symptoms and severity [26]. However, surgical correction has been shown to have higher success with relief of symptoms and achievement of patient goals than non-surgical options [27]. Over their lifetimes, approximately 12–19% of females will have surgery for POP [28,29]. Traditionally, surgery for prolapse involving the uterus was treated with vaginal hysterectomy (i.e., removal of the uterus) and upward suspension of the vagina using sutures [30]. The male surgeons who first developed hysterectomy techniques viewed the uterus as having a single biologic function, to carry children. Once this stage of life was complete, surgeons viewed uterine removal as the preferred way to treat uterine prolapse [31], an inherently patriarchal notion which grossly undermines the uterus’ value beyond childbearing.

Although surgical treatment for POP has evolved, with new methods of hysterectomy and uterine-preservation becoming available, surgical treatment for POP can be difficult to obtain, similar to women’s experiences with other female-specific disorders (e.g., endometriosis [32]). Physicians may be unaware of or ignore symptoms of prolapse, disregarding women’s discomfort, pain, and disability due to prolapse, and ultimately overlook their opinions and preferences for treatment [33]. Furthermore, the expectations and responsibilities of women in their daily lives may impact their decision-making for POP surgery, given the time commitment required for recovery and the need for support to meet women’s expected daily tasks.

### 1.3. Prolapse & Childbearing

Childbearing is a significant risk factor for POP. Pelvic floor disorders, including prolapse, are more common in parous women compared with nulliparous women [34,35,36], with increasing parity being positively associated with prolapse [37]. Additional obstetrical factors associated with increased risk of pelvic floor disorders include prolonged second (pushing) stage of labour, fetal macrosomia, and perineal lacerations [38]. Vaginal childbirth [39,40] has also been identified as a risk factor for pelvic floor disorder, given the physiological changes that occur to the pelvic floor during pregnancy, labour, and delivery. Women undergoing spontaneous vaginal delivery are 5.6 (95% CI: 2.2–14.7) times as likely to experience prolapse compared to women who undergo caesarean section [40]. Furthermore, the use of traction devices to assist in operative vaginal delivery (e.g., forceps, vacuums) significantly increases the risk of pelvic floor disorders [36,37].

### 1.4. Prolapse & Sexuality

In general, women with POP report being physically able to have sexual intercourse, despite the presence of a bulge; however, given the nature of prolapse and its impacts, women’s sexual function can be affected [41]. Evidence in this area is mixed and may be dependent on women’s specific symptoms of and severity of prolapse, with some women reporting minimal impact of prolapse on sexual function and other women reporting decreased sexual arousal, infrequent orgasm, and dyspareunia [42].

Moving beyond biomedical impacts, some studies have found evidence of the impact of prolapse on women’s body image [43]. Shah and colleagues [44] found that women with prolapse reported reduced sexual initiation and sexual function related to their self-image and self-esteem, which they attributed to prolapse. Sexual satisfaction, including orgasm, also decreased with the experience of prolapse. The decrease in sexual function also impacted women’s relationships with their partners and caused significant anxiety and distress.

### 1.5. Gender and Treatment Decision-Making

Patients are assumed to be able to navigate the range of options available to them for treatment and rationally select the best option for their lives in an autonomous fashion [45]. However, for women pursuing surgery for POP, these decisions are couched in cultural expectations of womanhood and femininity [31,46]. For some women, identity may be impacted by the loss of the uterus through hysterectomy, as the uterus is linked with femininity, sexuality, and motherhood; identities are further impacted given the value placed on motherhood by society [46]. Even without the loss of their uterus, the decision to have surgery for prolapse is complex. Thus, the lived experiences of women with POP add layered complexities to the presumed “rational” decision-making, and the intersection of gendered expectations may mean that decision-making does not center on the woman, but rather children, partners, and/or societal norms.

### 1.6. Current Study

Women’s healthcare decision-making is far from straightforward, influenced by relationships, moral obligations, and gender related identity [45]; however, there is a distinct lack of qualitative data on how women’s gendered expectations and norms intersect with POP and seeking surgery. Given the prevalence of prolapse and the lifetime risk of prolapse surgery, there is a significant need for research using a gender-based lens. Thus, this study aimed to explore how women’s gendered responsibilities and identities impact their experience of prolapse and their decision-making around surgical treatment for POP.

## 2. Materials and Methods

We conducted qualitative, one-on-one, semi-structured interviews among a subsample of women from the Hysterectomy versus Uterine Preservation for Pelvic Organ Prolapse Surgery (HUPPS), a prospective surgical registry of surgical and patient-reported outcomes for women seeking surgery for POP. Women were eligible to participate if they were at least 18 years of age, had a POP of stage 2 or more as diagnosed by the Pelvic Organ Prolapse-Quantification (POP-Q) system [47], demonstrated apical prolapse (i.e., the descent of the uterus, cervix, or vaginal vault), elected for surgical management of their POP, had no prior hysterectomy, desired no further pregnancy, and could communicate in English.

Women were elected for surgery after consultation with a Female Pelvic Medicine and Reconstructive Surgery (FPMRS) specialist. Consultation involved a detailed medical history, clinical exam, and discussion of potential treatment options, including either hysterectomy and vaginal vault suspension (i.e., surgery to fix a vaginal wall prolapse) or uterine suspension (i.e., the uterus is retained, and supported and secured to ligaments within the pelvis). Clinical discussions were guided by standardized information from the International Urogynecology Association and relevant clinical evidence.

Participants were recruited via email before their surgery from a sample of women who were scheduled for surgery from February to July 2021, if they had completed the baseline questionnaire and informed consent form for the larger study. If women chose to participate, then they were given the choice of interview via phone or Zoom. Interviews took approximately 45 min and were audio-recorded and transcribed verbatim. Interview questions focused on women’s experiences of living with prolapse, what brought them to seek surgical management of their prolapse, and their decision-making regarding surgical choice (i.e., hysterectomy-based, or uterine-preserving surgery). The interview guide is available in Appendix A Women were purposively sampled, based on age and surgery type, to allow for diversity in experiences and information-rich data [48]. Interviews were conducted by EK and KR. Sampling was deemed to be complete once data saturation was achieved, defined as no new information being obtained from the interviews and analysis [49,50].

### 2.1. Sex- and Gender-Based Analysis

Sex- and Gender-Based Analysis (SGBA) is a process of examining sex, gender, diversity, and equity within research, with the aim to identity factors contributing to health disparities and to promote equitable policy and practice [51]. SGBA acknowledges the importance of the impacts of gender and gender inequality on health, recognizing gender differences in power, privilege, and opportunity. To achieve SGBA in qualitative research, a feminist standpoint epistemology was applied to interview guides and analysis, acknowledging the context and perspectives of the individuals generating the questions, conducting the research, and interpreting the data [52,53]. Acknowledging that the researchers’ perspectives can never been fully eliminated from the research process, we assembled a team of all female researchers from various life-stages and disciplines so that participants’ experiences were understood in the “voices” of women [54] with the goal of advocating for social change [53].

### 2.2. Data Analysis

The qualitative data were analyzed using the Framework Method [55,56]. Firstly, transcriptions were reviewed to familiarize the researchers with the data. Secondly, using the interview guide and identified themes, KR and EK constructed a thematic framework for analysis. This framework was refined as new themes emerged. Thirdly, the final thematic framework was systematically applied to the transcriptions, with KR and EK coding themes to the thematic framework. Multiple themes could apply for a single passage. Fourthly, data from the thematic framework was charted. Each theme was extracted across all respondents, with KR and EK synthesizing the information for each theme. Finally, all researchers (KR, EK, EAB, NVS, and AD) discussed the final results and interpreted the findings, providing investigator triangulation from the fields of sociology, epidemiology, and clinical medicine [57].

## 3. Results

In total, 26 women participated in the interviews. Most women were White, with two women self-identifying as mixed race/ethnicity or as a woman of color. All participants reported their gender identity to be that of a woman. All reported being heterosexual except one woman who reported being bisexual. The average age of participants was 53 years (IQR: 40–67). Most women were married or in a common-law relationship (88.5%), with the others being widowed or separated. The median number of children born to participants was 2 (IQR: 2–3), with no women reporting nulliparity. Most women had completed at least some post-secondary education. In the sample, 54% of women opted for a hysterectomy-based surgery and 46% opted for a uterine-preserving surgery. Specific demographic and surgical characteristics of the participants is available in Table 1.

Three gendered themes emerged related to participants’ identities as women, mothers, and partners where women discussed the influence of each of their roles and responsibilities on their experience of prolapse and their treatment decision-making for prolapse.

### 3.1. Identity as Woman: Femininity, Societal Expectations, and the Uterus

#### 3.1.1. Prolapse & Femininity

Compared to proximal symptoms (i.e., the biomedically defined and experienced symptoms of prolapse), the distal impact of symptoms refers to the wider reverberations these symptoms have on women’s lives. These include the activities and routines that had to be forgone or modified—lest they incite pain, discomfort, or embarrassment—and the toll this took on women’s sense of self-worth, role fulfillment, and relationships. Both distal and proximal symptoms impact one’s QOL, but it appears to be through distal impacts that symptoms were weighed against the potential outcomes and inconvenience of surgery. Women often reported, explicitly or implicitly, that prolapse had affected their ability to fulfill their expectations of what a woman should be. Participants discussed the influence of prolapse and the embodiment of prolapse symptoms on their feeling of being a woman. Although some women noted that prolapse did not affect their identity as a woman, it still affected their perception of self and their femininity.

*“I’m still a woman and I’m still quite feminine but...it’s humiliating. It’s not that I felt like less like a woman, but you do [feel less womanly due to prolapse], you just do.”*

(P19)

Many women indicated that the nature of prolapse was not feminine and, thus, affected the way that they felt about their bodies.

*“Even if someone tells you that [you’re attractive], it doesn’t make up for feeling gross. And I know that it’s not just in my head, right? It’s not like I just don’t like the way it looks. It’s the fact that it’s hanging out.”*

(P2)

The embodied experience of prolapse also led women to feel like their bodies were “broken” and not working as intended. Women discussed the experience of prolapse, knowing something was not right with their bodies. Many women discussed the psychological impact of these symptoms including shame that their bodies did not work as they wanted.

*“I felt broken for quite a while, actually. And it was hurtful. When your own body doesn’t like you, it just sucks. Like, I’m in a dinner party and everything shoots out and… [people are] like, “Oh my God, what’s wrong?” And I’m like, “Oh, I just got a cramp.” I can’t tell them. So, my self-worth definitely took a little bit of a beating as a woman.”*

(P1)

Overall, prolapse affected women’s perceptions of their bodies and highlighted the difference between their ideas of what their bodies “should be” compared to how they existed with prolapse.

#### 3.1.2. Prolapse & Societal Expectations

Women also talked about the societal expectations of women and the impact of prolapse on being able to meet those expectations. Prolapse affected women’s ability to do housework (e.g., cooking, cleaning), to take care of their children, and to live their daily lives without discomfort. Many of the women discussed having to deal with discomfort and pain to meet the requirements of daily life because they felt like there were things that “must” get done.

*“I limit my exercise to the point where I start to sweat, and then I have to stop… Because I also have to cook, and I also have to clean. Those are activities that I have to get done.”*

(P17)

Some women felt shame that they were not able to meet their ideas of what women should be, and this affected their thoughts regarding prolapse surgery. Many women conveyed a sense of worry about the recovery time because they did not think that they would actually be able to rest for the amount of time necessary to recover.

*“[The doctor said] you need to do nothing [in recovery]. I almost felt like she was shaking her finger at me, telling me, “Don’t do this and don’t do that,” you know? And I thought, she does know women, because as women, we will not be able to stop... We’re so used to doing things.”*

(P5)

Some women also mentioned the societal expectation of women’s suffering, noting that when they mentioned their prolapse symptoms to others or even to their family physicians, that the response was that prolapse is something with which women just have to live. Participants felt like prolapse was a taboo subject that they were not supposed to or able to discuss with others, leading to the perception that women’s health issues were not valued in society as women are supposed to just deal with the prolapse in silence.

*“When [my doctor] saw the prolapse, it was almost like a shrug and that’s the sacrifice a mum makes. And so, I didn’t get a referral from her for the pelvic floor clinic or any discussion about pelvic floor health.”*

(P14)

*“Well and I think that’s just a symptom of the way we treat women and women’s bodies, like they’re just to have babies, right? That’s what a woman’s body does, right?... But then who cares for the woman after they give birth?”*

(P2)

*“I think a lot of women just live with it and don’t talk about it, and they’re kind of ashamed to talk about pelvic organs. It would be nice, if like the stigma around that was gone, because I know a lot of women have the same symptoms as me.”*

(P10)

#### 3.1.3. Womanhood & the Uterus

Women were asked how surgical treatment and their decision to remove or suspend their uterus for POP treatment affected how they felt about themselves as a woman. Those who elected a hysterectomy did not indicate having attachment to their uterus and indicated that the removal of their uterus for a hysterectomy-based surgery would not affect how they felt about their femininity or womanhood.

*“A lot of people feel it’s part of womanhood. But I say—get rid of it. There’s no reason for it, and also I’ll have less chance of cancer for it. Take it out of there.”*

(P6)

However, other women felt that the removal of their uterus would affect their femininity, which caused them to opt for a uterine-preserving surgery rather than a hysterectomy-based surgery.

*“It’s just kind of how I thought of my uterus… It’s what makes [me] a woman.”*

(P15)

### 3.2. Identity as Mother: Childbearing, Sacrifice, and Motherhood

#### 3.2.1. Prolapse & Childbearing

Most women talked about the impact of childbearing on their bodies, with many indicating that they perceived childbearing to be the root cause of their prolapse. Some women experienced shame at experiencing prolapse related to childbearing, feeling as though it was a physical failure.

*“I feel like just a bit of a failure, really. Like I feel like, it should be OK to have babies and still be normal. After having a C-section, I already felt like a bit of a failure and then I end up with prolapse, too. It’s kind of like, OK, maybe something’s not normal in my body.”*

(P21)

Furthermore, the demands of motherhood often prevented women from being able to take care of their bodies in a way that would prevent or ameliorate the symptoms of prolapse. Women discussed the immediate postpartum period, where many women experienced the symptoms of prolapse for the first time, as a time when they were focused on ensuring that the baby was doing well and no one, including themselves, was focused on their health.

Women also discussed trying to maintain their mothering duties as a hindrance to non-surgical treatment of prolapse, as women found physiotherapy and pessaries to be time-consuming and inconvenient when they already had so much happening in their daily lives.

*“And then I did try the pessary, but that wasn’t going to be an option, just the understanding of how that works and how often I would have to take it out. And it would just add a level of inconvenience to my life that I just can’t handle.”*

(P11)

Women also reported that their mothering duties could worsen their prolapse. This often led to making accommodations to keep up with their children while still trying to reduce the extent of their prolapse symptoms (e.g., no hiking, taking it easy one day so they could do something with the kids the next). This also caused women shame and guilt because they felt like they were not living up to the ideals of good motherhood, which in turn was a factor in the decision to pursue surgery.

*“And I wanted to run with my kids, too… I was like, “Oh, you go ahead, I’ll catch up with you. I hope you don’t get hit by a car. Because, you know, I’m going to pee if I run too fast [laughs].” So, yeah, that was really a part of wanting to keep up with them, and yet feeling held back by this problem.”*

(P17)

#### 3.2.2. Prolapse & Sacrifice

Many women talked about the sacrifice of motherhood, both generally and specifically related to prolapse. One woman likened the experience of motherhood to the experience of prolapse:

*“It didn’t change immediately after [having kids]. I felt feminine… But then, as the years wore on, and I’m carrying babies, and I’m doing housework, and I’m nursing… And then you’re exhausted all the time, and then things start to sort of implode, literally and figuratively [laughs]. You really are just trying to get through. You’re exhausted... Honestly, it was like a physical manifestation of how I felt. It was like my uterus was being pulled out from under me. Everything was going to fall out. And yet, I still had to meet all the obligations and responsibilities that I put on myself by …having kids.”*

(P17)

Women expressed feelings of frustration after other conservative treatment options like pessaries and physiotherapy did not resolve POP symptoms. Some women saw these as the logical first steps that were to be exhausted before considering surgical intervention, with women reporting that they did not want to get the surgery or that they had postponed their surgery because they did not want to take time away from their children or were worried about childcare during their recovery. Two women endured symptomatic prolapse for several more years than they would have liked by prioritizing their children above their own health, as they postponed their surgeries until their children were older and did not need them as constantly.

Some women also felt as though even the decision to get surgery was “selfish”, due to the decision to accept the risk of surgery when it was something that they could “live with”, thinking of potential complications from the procedure and time for recovery. The role of motherhood caused women to put their children’s well-being first and their own self-care last, which played into the societal expectation of women and of prolapse being a burden that women just silently bear.

*“I struggled with taking this risk at all because my kids are young and there’s always risk of complications. It was hard to [say] it’s OK to go for this to improve quality of life and to take this risk. It felt selfish, I guess, for me. And so it took a while to get more comfortable with that feeling of it’s OK to prioritize [the surgery].”*

(P14)

Overall, women endured the symptoms of prolapse and increasing prolapse severity as a sacrifice to their roles as mothers. For many women, pain was a defining feature of POP. In addition to pelvic pain, participants reported lower back pain that sometimes extended to the legs. Such pain was often accompanied by unpleasant feelings of heaviness and pressure in the pelvis. Some women’s experience with pain was constant, but for the majority pain was situationally triggered by events like menstruation, sexual intercourse, protrusion or bulging of the POP beyond the vagina, or physical activity. Within the domain of physical activity, even low exertion activities like walking, lifting, or standing for prolonged periods of time were reported to trigger pain. Familial divisions of labor rendered many women unable to opt out of pain-inducing activities, as they often overlapped with women’s domestic and caregiving roles.

*“My husband’s working. My kids are online at school. And so, I gotta do what I gotta do. I just do it. I just pull through. It’s kind of hard to avoid the jobs at home that I have.”*

(P17)

While ceasing or reducing the frequency and duration of such activities in familial contexts did not incur the same financial penalty as professional accommodations, it was commonly accompanied by feelings of guilt, inadequacy, and moral failure. Though done for their own health, it was common for women to speak of these accommodations in evaluative, self-critical terms that centered the negative effect it had on their loved ones.

*“But we’re actually going to have to switch [my daughter] into a daybed, so I don’t have to lift her up and over. So, then there’s that evil... My prolapse is robbing my daughter of her childhood. I have to put her in a big girl bed because I can’t lift her up”.*

(P15)

#### 3.2.3. Motherhood & the Uterus

Women were asked about their feeling of their uterus and how it affected their decision-making around surgery. All of the participants had completed their childbearing, so many women (especially women of older age) indicated that they did not feel like they “needed” their uterus anymore as they would not be having any more children. These women tended to opt for hysterectomy-based surgeries. However, other women had more attachment to their uterus such that even if they were done having children, they still felt that the uterus represented their reproductive potential and affected their connection to motherhood.

*“[The thought of losing my uterus] made me angry. It made me feel like I wasn’t going to be as much of a woman anymore. And that’s so stupid. I know it. The thought of losing the organ...the home of my babies…I would definitely say it’s affected me.”*

(P15)

### 3.3. Role as Partner: Sexuality & Confidence

#### 3.3.1. Prolapse & Partnered Sexuality

Prolapse had a significant impact on women’s sexuality and sexual function. Some women felt that the experience of prolapse did not hinder their sex lives with their partners, but others mentioned that they experienced sexual discomfort or would avoid sex with their partners because of their symptoms of prolapse. Women often felt frustration or guilt because they perceived that they were not meeting their partner’s needs around sex.

*“And my sexual relationship with my husband, poor guy. I don’t want to have sex, right?... I didn’t feel good in my body. And psychologically, I think it was it’s been messing with me too.”*

(P2)

Women noted that they often had to be careful about engaging in sexual activity, making accommodations for types of sexual activity or sexual positions. Some women mentioned that they might avoid sexual activity on certain days of the month or after long days of physical activity.

*“So, it’s been life as normal [except for] the fact that sex has become more and more painful, and more so after sex... if sex is actually good, then I’m in pain for days. So that just became less and less.”*

(P7)

Some women mentioned that their partners were understanding of their prolapse and its impact on their sexual relationship. Other women mentioned that they did not feel comfortable talking to their partners about the prolapse and would try to hide the symptoms from them, feeling that this was not an attractive quality to present to their sexual partner. Although it was not the primary reason for seeking surgery, many women reported that they wanted their sexual function to go back to normal after surgery and this was a factor in seeking surgical treatment.

Even though many of the women indicated that their partners did not seem to be affected by the prolapse, women were often still hesitant to engage in sexual activity. Women were embarrassed by the prolapse and its symptoms, being worried about incontinence during sexual activity, or their husband noticing the bulge.

*“I mean, there are times where it really does stick out, you know, it’s right there. And so, yeah. That’s for me personally, mentally off-putting. [Laughs] For me, I can’t imagine how anyone else would, you know, find that interesting.”*

(P17)

Many women noted that the issues relating to sexual activity were more psychological than physical, with their confidence around sexual activity being affected by the prolapse.

*“I feel less confident about how I how I look and how things feel down there. And my husband assures me that that is not the case for him.”*

(P14)

#### 3.3.2. Feeling Guilt or Shame about One’s Sexual Response to Their Prolapse

In discussions of sexuality, many women reported feeling guilty or ashamed that they had decreased sexual activity due to their prolapse. Many women had sex less frequently than they did pre-prolapse, due to pain, decrease in sensation, or matters of self-esteem. The loss or reduction (in quality and/or frequency) of “good sex” was something younger women mourned and described as affecting their QOL. However, women did not conceive of their sexuality in a vacuum and regularly expressed feelings of moral responsibility to their partner. Sometimes these considerations of their partners’ loss were foregrounded and internalized as a failure to live up to one’s relational role requirements.

*“How can I be a wife anymore? How can I be a woman anymore if I cannot function this way? … This was one of the things I just didn’t know how to handle anymore, and I knew surgery would fix it.”*

(P25)

## 4. Discussion

This qualitative study described the experiences of 26 women seeking surgical treatment for POP, finding that women’s decision-making for prolapse surgery was complex, affected by their gendered responsibilities and identities as women, mothers, and partners. These three thematic identities are unique, yet overlap, and highlight the multiple roles that women must balance with their own health and how they consider these roles and responsibilities as they navigate treatment-seeking for prolapse.

We found that the experience of prolapse is highly nuanced within women’s views of femininity, motherhood, and being a “broken” woman. This is not surprising given the pervasive and ingrained societal messaging that bodily impairments are inherently negative [58,59]. While physical deviations from the norm may be tolerated, society has subtly signaled to women that bodily changes result in a state of being less than [58,59]. When coupled with society’s patriarchal belief that even “normal” femaleness is inherently inferior [60], it is no surprise that women with POP felt “broken” and a sense of pressure to normalize their bodies [60].

Women’s decision-making around which surgical treatment to pursue, the timing of said treatment, and their future outlook was contextualized by gender norms. Prolapse symptoms seemed to plague their perceptions of what makes a “good” woman, “good” mother, or “good” partner, with women perceiving that their prolapse affected their ability to live up to the societal standards of these roles, aligning with the ideology of “intensive mothering” where “good mothers” should be caregivers who invest all of their available resources into their children [12]. This motivated women to address their prolapse permanently (i.e., through surgery) so that they could return to these roles or live up to the gendered expectations of these roles. Overall, women experiencing prolapse seemed to try conservative options for treatment first, as these would be the least disruptive to their lives and that of their family. Other studies of women with prolapse have reported parallel findings, with women having limited access to healthcare or information about their health issue, fewer healthcare options, and prioritizing the health of their children and partners above their own health [11,33,61,62].

When conservative treatments failed, women weighed their symptoms and the likelihood of the progression of these symptoms with their other responsibilities, recognizing that choosing a surgical option for prolapse treatment would mean that they would not be able to meet their perceived responsibilities during their recovery. This gave many women pause, with some women postponing surgery until they could pass these responsibilities to others or until their responsibilities were lessened. This highlights the psychosocial context in which women find themselves when considering non-urgent treatment, even that which will significantly improve their QOL. In general, women are expected to sacrifice their time, health, and bodies for their children and partners, at the expense of themselves. Women have the tendency to put their time and available resources into their household, with this being viewed as a “badge of honor” and being socially sanctioned, aligned with the notion that the welfare of the family is primarily the responsibility of the woman [63]. This normalizes the expectation that women should make sacrifices in pursuit of her family’s welfare.

Like our findings, a recent study exploring the social construction of choice for women living with breast cancer found that treatment choice for women is “layered in the gendered moralities and ethics of care (and obligation)” [45] (p. 6). The authors discussed how women must balance familial responsibilities, normative discourses of being a “good” patient, and their own needs and wishes, highlighting the interrelation between women’s decisions to care for themselves and to care for others [45]. In our study, women’s desire for surgery was disrupted by their responsibilities as a caregiver to children but promoted by their responsibilities as a partner and identity of a woman. These intersections forced women to reconcile their need for treatment even if it was at odds with their role as caregiver and partner.

Women’s bodies have not been made a health priority [64], especially beyond their utility in childbearing and as sexual partners. Furthermore, our results demonstrate the lack of societal value placed on older women’s reproductive health. Given that prolapse often occurs decades after childbearing has been completed, combined with the time accrued as women convinced themselves that it was permissible to prioritize themselves, the women in our study often suffered in silence for years. Because prolapse impacts both the physical and mental health of women, this period of nontreatment is concerning given that maternal health has potential for lasting intergenerational impacts [65]. POP surgery has been associated with significant improvements in health-related QOL [66]; thus, promoting access to treatment for women experiencing prolapse would likely have positive impact that extends beyond the affected individual, to her partner and children.

Women are often responsible for the invisible labours of childcare and housework, in addition to paid labours outside the home [67], resulting in barriers for women to access healthcare in general [64,68]. These gender-based barriers were also described as specific to POP treatment in our study, as women expressed their concern regarding surgical recovery time, which inhibited their treatment-seeking for years due to their household responsibilities. Furthermore, women highlighted barriers to treatment in the healthcare system due to physicians’ attitudes and beliefs about POP being an acceptable consequence of childbearing. It has been previously documented that many physicians are unaware of the symptoms of prolapse [33,69] and put together with the minimization of women’s symptoms, our study raises concern that institutional barriers within medicine exist for those seeking POP treatment, which leads to a lack of referral to specialized care and the continued suffering of women until symptoms are unbearable. In Alberta, where this study was conducted, prolapse surgery is currently covered under the public healthcare system, meaning that women are not required to pay directly for the expenses related to their surgery. As of 2021, however, prolapse surgery is under review by the provincial health system as a “low-value” procedure to no longer receive funding [70], which ignores the evidence that prolapse impacts women’s everyday lives, ability to work, and QOL [66]. Our study goes beyond these traditional metrics of medical value, by showing that POP also impacts the societal constructs of gender identity and roles. This potential defunding of POP surgery in Alberta is of serious concern, as making prolapse surgeries private pay would create significant barriers to surgical treatment as women would have to pay out-of-pocket, a prohibitively expensive proposition leading to further inequities. If successfully defunded in one province, concern would arise that other provinces may follow suit in years to come, impacting not only those in one province but all Canadian women.

To our knowledge, this is the first qualitative study to examine women’s treatment decision-making for POP from a gender-based lens, highlighting women’s voices and centering their experiences and responding to the call for a more holistic approach to women’s health [71]. Given that there is a dearth of qualitative literature examining the experiences of women with POP and their decision-making around surgical treatment, our research adds significant depth to this narrative. This study also heightens awareness of prolapse as a women’s health issue, helping to reduce the stigma and shame that women feel, normalizing treatment-seeking for POP and conveying the need for information about POP to become part of society’s discussion regarding health. This is an important area of research to inform equitable health and healthcare policy and practice development, as women’s health issues are often marginalized, trivialized, and are often viewed within the limits of a biomedical lens [71,72,73]. With the emergence and recognition of the importance of patient-oriented care, research and policy in this area must continue to engage women directly to understand the complexities of their experiences, the barriers that they face in seeking care, and the implications of surgical treatment on their lives.

One limitation of this study is that we focused specifically on women who were already seeking treatment for prolapse and who had opted for surgical management of their prolapse symptoms. It is likely that certain groups of women were not represented due to health inequities preventing them from seeking care in the first place (e.g., some women may not seek care because of structural issues like discrimination, racism, inability to take time off work). Future research should examine the barriers that may prevent women from seeking surgery or other treatment options for POP. Furthermore, the study sample was fairly homogenous, with few women identifying as members of minority groups and most women reporting relatively high socioeconomic status. The phenomenon of White, middle-class women being the voice of women’s research is not a new concern [74,75] and given that we recognize that gender intersects with other phenomena, such as race, ethnicity, and sexual orientation, future research is needed to examine inequities in access to gynecological care with a focus on intersectionality [76,77].

## 5. Conclusions

This study examined the intersection of POP and women’s gendered expectations and responsibilities, demonstrating the gendered inequities existing for women in their experiences of POP and its treatment. Prolapse and identity were reciprocal in nature, with prolapse affecting participants’ identities as women, partners, and mothers and these identities influencing their experience of prolapse. Women faced barriers to treatment related to their expected roles and responsibilities as partners and mothers, coupled with a lack of awareness around and prioritization of prolapse by their healthcare providers. Findings highlight women’s experiences of prolapse in the context of their healthcare needs and can inform policies and practices which promote more equitable access to prolapse treatment.

## Figures and Tables

**Table 1:** Demographic and Surgical Characteristics of Participants.

Participant Number	Surgery Type	Age	Ethnicity	Partner Status	Parity	POP-Q Stage	SUI Procedure
1	Hysterectomy	54	White	Married	2	2	Yes
2	Hysterectomy	41	White	Common-law	3	2	Yes
3	Hysterectomy	60	White	Married	2	2	No
4	Hysterectomy	60	White	Married	2	2	No
5	Hysterectomy	75	White	Married	3	2	Yes
6	Hysterectomy	49	White	Married	2	2	No
7	Hysterectomy	41	White	Common-law	2	2	No
8	Hysterectomy	54	Black	Married	3	3	No
9	Hysterectomy	75	White	Widowed	4	3	No
10	Hysterectomy	31	White	Married	2	2	No
11	Hysterectomy	40	White	Married	2	2	Yes
12	Hysterectomy	68	White	Married	1	3	No
13	Hysterectomy	71	White	Married	3	2	Yes
14	Hysterectomy	40	White	Married	2	2	No
15	Uterine Preservation	32	White	Married	3	2	Yes
16	Uterine Preservation	63	White	Separated	2	2	No
17	Uterine Preservation	52	Mixed	Married	2	2	No
18	Uterine Preservation	51	White	Married	2	2	No
19	Uterine Preservation	67	White	Married	2	2	Yes
20	Uterine Preservation	73	White	Married	3	4	No
21	Uterine Preservation	40	White	Married	3	2	No
22	Uterine Preservation	74	White	Widowed	1	3	Yes
23	Uterine Preservation	34	White	Married	2	*	*
24	Uterine Preservation	39	White	Married	2	2	No
25	Uterine Preservation	45	White	Married	4	2	Yes
26	Uterine Preservation	60	White	Married	3	3	No

* Not reported as participant’s surgery was cancelled due to COVID-19 restrictions and has not yet been completed.

## Data Availability

Data used for this study are not publicly available.

## Appendix A. Semi-Structured Interview Guide

1. **I understand you are scheduled/have recently had surgery for pelvic organ prolapse with a surgeon at the Foothills Hospital. Could you describe how you came to this point?**
  Prompts:
  - *What led you to seek treatment? In what ways has prolapse impacted you?*
  - *Was your prolapse something you “discovered” on your own, or did a health professional diagnose it first and then tell you that you had it?*
  - *What types of care providers have you consulted in the process? What did those care providers tell you about prolapse in general and how it can be treated?*
  - *What was the length of time between first “discovering” your prolapse and seeing your surgeon for the first time? How long did you wait for surgery? Did you think that was a reasonable wait?*
  - *What has the experience of the condition been like for you? How has it affected your daily life, including activities and relationships?*
  - *Has your condition had an impact on your intimate relationships or sex life?*
2. **Thinking back to before you had your first visit with your surgeon, do you remember having any thoughts or opinions about what would or should be done about your POP?**
  Prompts:
  - *If the woman had some pre-existing thoughts/opinions, ask what experiences she thinks lead to those opinions (e.g., family member, friend, internet, etc.)*
  - *Prior to that visit, were you specifically seeking surgery, or hoping to avoid it?*
3. **Prior to those medical visits, were you aware that sometimes the uterus is removed as part of surgery to repair prolapse?**
4. **Is this the decision you thought you were going to make before you saw the surgeon?**
5. **Please think back to the consultation and any follow-up visits you had with your surgical team. How did your consult with your surgeon affect your thinking about your condition and its treatment? What did you take away from that consultation as the most important considerations?**
  Prompts:
  - *Were the options of having your uterus removed (hysterectomy) versus having the uterus suspended in a thorough and clear manner? Were you left with any questions that were unanswered?*
  - *Do you feel like any of the information the surgeon presented was conflicting? Or confusing?*
  - *Were you surprised at all by the information they gave you?*
  - *Do you feel like the way you were counselled was prescriptive—such as “you should do this….”—or were you the one to make the choice about hysterectomy vs. uterine suspension—such as “I will do either, whichever you would like”*
  - *Do you feel like the surgical advice about what to do with your uterus was specifically tailored to you—such as “based on your PAP smear results, or menstrual pain, I would recommend XYZ”*
6. **What is your understanding of the treatment options (hysterectomy or uterine suspension as part of your prolapse repair) that are available to you?**
  Prompts:
  - *What do you understand as the pros and cons of these treatment options?*
  - *Was one of the options—hysterectomy vs. uterine suspension—presented has having more or less surgical risk? More or less surgical success?*
7. **Is the surgical choice you made—hysterectomy or uterine suspension—the decision you thought you were going to make before you saw the surgeon?**
8. **Did the diagnosis of pelvic organ prolapse change how to you viewed yourself as a woman?**
9. **Would removal of your uterus as part of a surgical procedure change how you viewed yourself as a woman?**
  Prompts:
  - *Do you feel as through removal of your uterus is inherently tied to your gender identity?*
  - *If under age **45**, would loss of your reproductive potential through removal of the uterus change how you view your overall health or “womanhood”?*
  - *If under age **50**, does the loss of your menstrual cycle through removal of your uterus change how you view your overall health or “womanhood”?*
10. **Have you talked to anyone other than your surgeon about this decision? If yes, did that play a part in your decision?**
11. **What other sorts of information have you consulted to try to understand your condition and treatment options?**
12. **What do you hope the results of the surgery will be? What kind of results from the surgery will make it worth undergoing surgery, in your mind?**
